# Prognostic Factors in Staged Bilateral Total Knee Arthroplasty—A Retrospective Case Series Analysis

**DOI:** 10.3390/jcm12103547

**Published:** 2023-05-18

**Authors:** Krystian Kazubski, Łukasz Tomczyk, Andrzej Bobiński, Piotr Morasiewicz

**Affiliations:** 1 Department of Orthopaedic and Trauma Surgery, University Hospital in Opole, Institute of Medical Sciences, University of Opole, Witosa 26, 45-401 Opole, Poland; 2 Department of Food Safety and Quality Management, Poznan University of Life Sciences, Wojska Polskiego 28, 60-637 Poznan, Poland

**Keywords:** total knee replacement, two-stage, prognostic factors, bilateral, predictive factors

## Abstract

Background: Bilateral osteoarthritis of the knee is an indication for a bilateral total knee replacement (TKR) procedure. The goal of our study was to assess the sizes of the implants used during the first and second stages of TKR procedures in order to compare their size and identify the prognostic factors for the second procedure. Methods: We evaluated 44 patients who underwent staged bilateral TKR procedures. We assess the following prognostic factors from the first and second surgery: duration of anesthesia, femoral component size, tibial component size, duration of hospital stay, tibial polyethylene insert size, and the number of complications. Results: All assessed prognostic factors did not differ statistically between the first and second TKR. A strong correlation was found between the size of femoral components and the size of tibial components used during the first and second total knee arthroplasty. The mean duration of the hospital stay associated with the first TKR surgery was 6.43 days, whereas the mean duration of the second hospital stay was 5.5 days (*p* = 0.211). The mean sizes of the femoral components used during the first and second procedures were 5.43 and 5.2, respectively (*p* = 0.54). The mean sizes of the tibial components used during the first and second TKR procedures were 5.36 and 5.25, respectively (*p* = 0.382). The mean sizes of the tibial polyethylene inserts used during the first and second procedures were 9.45 and 9.34 (*p* = 0.422), respectively. The mean duration of anesthesia during the first and second knee arthroplasty was 117.04 min and 118.06 min, respectively (*p* = 0.457). The mean rates of recorded complications associated with the first and second TKR procedures were 0.13 and 0.06 per patient (*p* = 0.371). Conclusions: We observed no differences between the two stages of treatment in terms of all analyzed parameters. We observed a strong correlation between the size of femoral components used during the first and second total knee arthroplasty. We noted a strong correlation between the size of tibial components used during the first and second procedure. Slightly weaker prognostic factors include the number of complications, duration of anesthesia and tibial polyethylene insert size.

## 1. Introduction

Total knee replacement (TKR) procedures are an important proportion of all orthopedic surgeries worldwide [1,2], with approximately 1.5–2 million total hip replacement (THR) and TKR procedures in the United States being performed annually [1,2,3]. An estimated 2.34% to 4.55% of individuals aged 50 or more have undergone a total hip or knee replacement surgery [2].

Bilateral osteoarthritis of the knee is an indication for a bilateral TKR procedure [4,5,6,7,8,9,10,11,12,13,14,15,16,17,18,19,20,21]. Approximately 19–30% of patients with degenerative joint disease of the knee require bilateral total knee arthroplasty [8,11,17].

There are two management strategies available for patients diagnosed with bilateral knee osteoarthritis: simultaneous bilateral TKR or a staged treatment involving two consecutive TKR procedures performed one at a time [7,8,9,10,11,12,13,14,15,16,17,18,19,20,21]. Many orthopedic surgeons consider either treatment strategy to be beneficial [8,9,10,19]. However, most authors choose the staged approach, which reduces loss of blood, the rate of complications, the extent of the procedure, and the required rehabilitation period and enables the patient to more rapidly resume physical activity [8,9,10,11,16,17,20]. Another potential advantage of the staged approach over simultaneous TKR is the opportunity to determine the prognostic factors for the second procedure [7,11,17,22].

Neither the postoperative symmetry of endoprosthetic parameter assessment following bilateral TKR procedures, nor the assessment of associated prognostic factors, has been extensively investigated, and literature data on these subjects are sparse [7,9,11,17]. To date, authors have compared the staged and simultaneous TKR procedures only in terms of the main complications and treatment outcomes [8,9,10,11,14,15,20,21]. Approximately 20% of patients following a unilateral TKR procedure are dissatisfied with the treatment and decide to forego the procedure in the other knee [17]. Therefore, it is imperative to identify the prognostic factors and assess the risk of complications for the second procedure. There have been no studies in which the data from the first TKR procedure were used to analyze the prognostic factors that could affect the subsequent procedure in the contralateral knee. A thorough understanding of the various factors involved in and resulting from single knee arthroplasty may considerably facilitate the course of the subsequent procedure in the other knee [7,11,22]. Knowing the parameters of the components already implanted during the first procedure (femoral component size, tibial component size, and tibial polyethylene insert size) may considerably facilitate the planning of the surgery for the other knee joint. This would also prepare the orthopedists for potential difficulties and complications, which would greatly improve the course of the procedure and the planning of rehabilitation [7,11,22].

In our study, we set two objectives: first, assess the sizes of the implants used during the first and second stages of TKR procedures; second, identify the prognostic factors for the second procedure in two-staged bilateral TKR procedures.

We hypothesized that the size of the implants used during the first and second stages of TKR procedures will be the same and that there will be a correlation between the parameters we evaluate during the first and second operations.

## 2. Materials and Methods

### 2.1. Study Design

This study was a retrospective case series analysis of TKR surgeries performed at a teaching healthcare facility that deals with comprehensive diagnostics, surgical treatment, postoperative follow-up, and rehabilitation.

### 2.2. Patients

In the period between 2017 and 2021, 50 patients underwent staged bilateral TKR procedures. All 50 patients were operated on due to advanced bilateral osteoarthritis of the knee and the associated severe pain, in the absence of improvement after the use of rehabilitation, analgesics, symptomatic slow-acting drugs for osteoarthritis (SySADOA) and lifestyle modification. Study inclusion criteria were a staged bilateral TKR procedure due to knee osteoarthritis, complete medical records, and complete radiographic data. The exclusion criteria were a unilateral TKR procedure, unicompartmental knee arthroplasty, distal femoral osteotomy or proximal tibial osteotomy, incomplete radiographic records, or incomplete medical records. The study was conducted in accordance with the Declaration of Helsinki, and the study protocol had been approved by the local ethics committee.

Six patients were excluded from the study due to the lack of complete radiological documentation. Once the inclusion and exclusion criteria were applied, a total of 44 patients (24 women, 20 men) were found to be eligible for our retrospective analysis. The mean age of those patients was 67 years (range 53–77 years). The TKR procedures in all patients were performed by one out of three experienced orthopedic surgeons. The staged procedure was performed by the same surgeon in the 1st and second surgery. The surgical technique (implant insertion and placement) was identical in all cases, and all patients had identical rehabilitation regimens.

### 2.3. Methods

We reviewed all medical and radiographic records in order to assess duration of anesthesia and hospital stay, femoral component size, tibial component size, tibial polyethylene insert size, and the number of complications (infection, prosthetic dislocation, delayed surgical wound healing, periprosthetic fracture, deep vein thrombosis, pulmonary embolism, hematoma, cardiac complications, respiratory complications).

We compared the first and second stage of TKR procedures in terms of all the evaluated prognostic factors. To identify the prognostic factors for the second surgery, we analyzed the correlation between the following parameters from the first- and second-stage procedure: femur implant size, tibia implant size, tibial polyethylene insert size, the duration of anesthesia, the duration of hospital stay, and the number of complications.

### 2.4. Statistical Analysis

Data were statistically analyzed using Statistica 13.1. The Shapiro–Wilk test was used to check for normality of distribution. The Wilcoxon signed-rank test was used to compare quantitative variables. A Spearman’s rank correlation coefficient was used to test the correlation between the variables. The level of statistical significance was set at *p* < 0.05.

## 3. Results

We analyzed the outcomes of staged bilateral TKR surgeries in 44 patients. In 29 cases, the knee endoprosthesis was implanted first on the right side. In 15 patients, the left knee was operated on first. The outcomes have been presented in Table 1, Table 2 and Table 3.

All assessed prognostic factors did not differ statistically between the first and second TKR. A strong correlation was found between the size of femoral components and the size of tibial components used during the first and second total knee arthroplasty. The mean duration of the hospital stay associated with the first TKR surgery was 6.43 days, whereas the mean duration of the second hospital stay was 5.5 days. This difference was not statistically significant (*p* = 0.211)—Table 1.

The mean sizes of the femoral components used during the first and second procedures were 5.43 and 5.2, respectively. This difference was not significant (*p* = 0.54)—Table 1.

**Figure 1:**
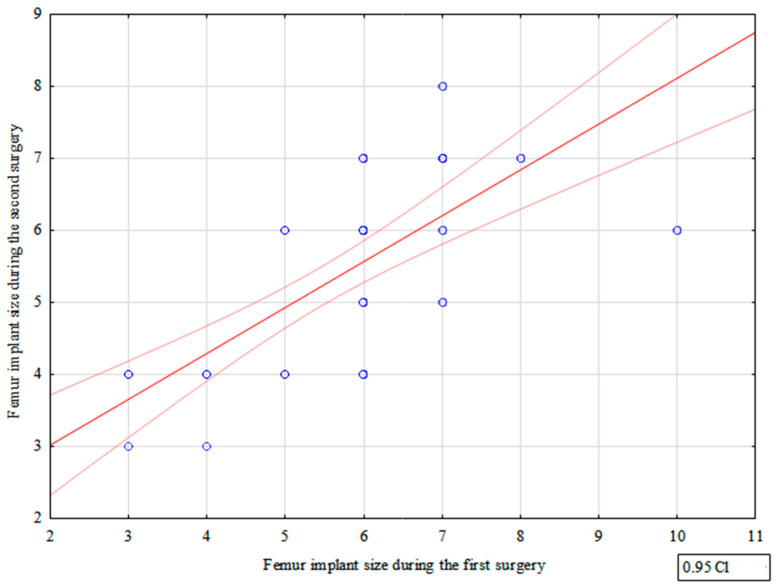
Correlation between femur implant sizes used during the first and second surgery.

We observed a strong correlation between the femoral component size used during the first and second TKR procedure (correlation coefficient = 0.790)—Figure 1, Table 2.

**Figure 2:**
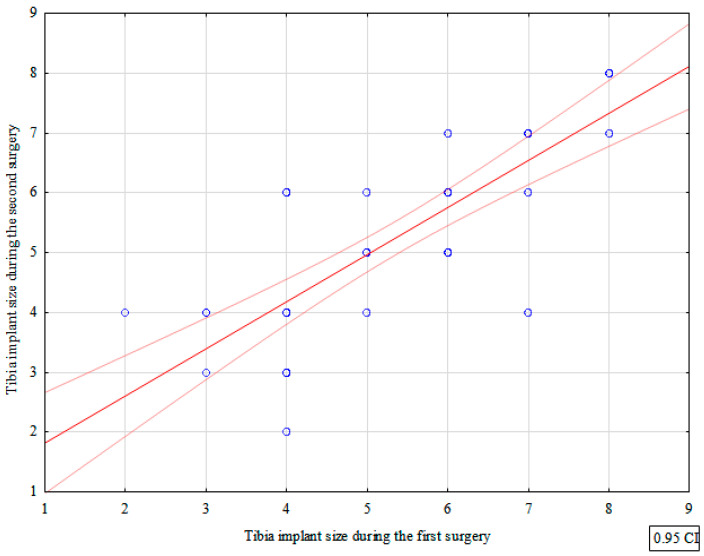
Correlation between tibia implant sizes used during the first and second surgery.

The mean sizes of the tibial components used during the first and second TKR procedures were 5.36 and 5.25, respectively. There were no significant differences between the two procedures in terms of the tibial component size (*p* = 0.382)—Table 1. We observed a strong correlation between the tibial component size used during the first and second procedures (correlation coefficient = 0.820)—Figure 2, Table 2.

The mean sizes of the tibial polyethylene inserts used during the first and second procedures were 9.45 and 9.34, respectively. These differences were not statistically significant (*p* = 0.422)—Table 1.

The mean duration of anesthesia during the first and second knee arthroplasty was 117.04 min and 118.06 min, respectively. The two procedures showed no significant differences in terms of anesthesia duration (*p* = 0.457)—Table 1.

The mean rates of recorded complications associated with the first and second TKR procedures were 0.13 and 0.06 per patient. This difference was not statistically significant (*p* = 0.371)—Table 1. The first and second procedures combined were associated with nine cases of delayed surgical wound healing (due to hematoma reabsorption). In each of these cases the wound swab cultures done during the hospitalization were negative, and C-reactive protein (CRP) and procalcitonin levels were within normal limits. There were no cases of surgical wound infection, prosthetic dislocation, deep vein thrombosis, periprosthetic fracture, pulmonary embolism, hematoma, cardiac complications, or respiratory complications, either during the first or during the second hospital stay.

## 4. Discussion

In our study, we found no statistically significant differences between the two stages of TKR in terms of the duration of anesthesia, duration of hospital stay, femur implant size, tibia implant size, tibial polyethylene insert size, or the number of complications. We observed a strong correlation between the size of femoral components and the size of tibial components used during the first and second total knee arthroplasty.

The main purpose of TKR procedures is to improve the range of motion and pain in the knee joint, and consequently improve the motor function of the lower limb [1,4,5,6,12,18]. TKR procedures often help the patients become more physically active and improve their quality of life. According to the available data, 19%–30% of patients require bilateral total knee arthroplasty due to bilateral knee osteoarthritis [8,11,17]. The opinions on the surgical approach to patients with bilateral knee osteoarthritis are divided [7,8,9,10,11,12,13,15,17,20]. Some authors prefer simultaneous bilateral TKR procedures [12,13,15], whereas others choose the staged treatment for bilateral knee osteoarthritis [8,9,10,11,17,20].

The staged approach to knee osteoarthritis treatment may be better than simultaneous bilateral knee arthroplasty due to the possibility of identifying prognostic factors for the second procedure [7,11,17,22]. It may be important to identify and predict the risk factors for the second surgery in patients undergoing bilateral TKR procedures. Bilateral TKR procedures have not been extensively evaluated, particularly in terms of assessing the symmetry of implant size in both limbs and identifying the prognostic factors for the second surgery [7,11]. Most authors have focused on comparing simultaneous and staged TKR procedures in terms of complication rates and treatment outcomes [8,9,10,11,14,15,20,21]. Assessing the patients who undergo staged bilateral TKR procedures will help better prepare for the second stage, identify risk factors, and plan further stages of patient treatment and rehabilitation. Moreover, it will help the surgeon prepare for possible intraoperative difficulties and complications, which will considerably improve the course of treatment.

Scott observed no correlation in the level of patient satisfaction associated with the first and second TKR procedure [17]. Wang et al. compared 12 patients who underwent unicompartmental knee arthroplasty during the first stage and total knee arthroplasty during the second stage and 12 patients who underwent staged total knee arthroplasty [7]. Those authors observed no clinical, radiographic, or functional differences between the evaluated groups [7]. Warren et al. analyzed the complications of simultaneous bilateral TKR procedures and staged treatment [9]. The authors observed a lower risk of complications in the staged surgery group [9]. Another study analyzed 39 patients following unilateral total knee arthroplasty and 36 patients following simultaneous bilateral total knee arthroplasty [10]. Those authors reported higher rates of complications and blood transfusions in the simultaneous bilateral total knee arthroplasty group [10]. In their systematic review and meta-analysis, Liu et al. evaluated 73,617 patients following simultaneous bilateral total knee arthroplasty and 61,838 patients following staged total knee arthroplasty [8]. Their analysis of various complications showed neither of the two strategies to be superior in terms of safety [8]. Grace et al. analyzed 36,278 patients who had undergone staged bilateral total knee arthroplasty [11]. These authors reported that all types of complications observed during the first procedure significantly increased the risk of complications during the second procedure [11]. Our study showed a moderate correlation between the rates of complications associated with the first and the second procedure. Ahd observed complications in 13.5% of total knee arthroplasty patients [18], which was a higher proportion than that observed in our study (10.2%).

It seems important to assess the possibility of predicting the length of stay during the second TKR operation, knowing the length of stay during the first TKR operation. The period of hospital stay after surgery is important for patients, doctors and hospital administration. When planning surgery and admission to the hospital, patients want to know how long the hospitalization will last, how long they will be away from home, how much stuff (e.g., clothes, food) they should bring to the hospital, etc. The doctor, knowing the estimated duration of stay, is able to better manage the movement of patients and the occupancy of beds in the ward, and can better calculate the costs of treatment. The hospital administration, knowing the estimated duration of stay, can more accurately predict the cost of treatment and the staffing of doctors and nurses in the ward. The period of hospital stay in the population evaluated by Wang was 7.9 days [7] and in that evaluated by Ahd—12.7 days [18]. In a group of patients after TKR from Italy, the average period of hospitalization was 8.1 ± 2.4 days [23]. The average length of hospital stay in the group of patients after TKR from Pakistan was 7 days [24]. Halawi reported an average hospitalization period of 3 days among a group of patients from the United States after TKR [25]. In a group of Chinese patients after TKR, the average hospital stay was 8.3 days [26]. The period of hospitalization after TKR reported by other researchers [7,18,23,24,25,26] from different countries was similar to our results. Our study showed no significant differences between the first and second surgery of staged bilateral TKR procedures in terms of the duration of hospital stay. The correlation between the duration of the first and second hospitalization was weak.

In our study we observed no significant differences between the duration of anesthesia during the first and second procedure. There was a moderate correlation between the duration of anesthesia during the first and second TKR procedure.

We observed a strong correlation between the femoral component size during the first and second TKR surgery. Moreover, we noted a strong correlation between the tibial component size during the first and second procedure.

In our study, 29 patients had their right knee operated on first, and 15 patients had their left knee operated on first. The fact that 65% of patients first underwent right knee arthroplasty suggests a higher rate of degenerative changes in the right knee. Most of the evaluated patients had a dominant right lower limb, did physical labor, and were retired. It is possible that right lower limb dominance may have accelerated the development of degenerative chances in the right knee joint, in a similar way as that observed in the right hip joint [22]. However, the small sample size prevents us from drawing such conclusions and necessitates caution in data interpretation.

The limitations of our study were the relatively small sample size (44 patients), exclusive analysis of medical and radiographic records, and the retrospective nature of the study. Nonetheless, some other studies were also retrospective in nature [10,11,18,20,22] and involved populations of similar size [7,10,12,19,22].

The strengths of our study were the fact that the procedures were performed by one out of only three orthopedic surgeons with the use of the same surgical technique and had the same rehabilitation regimen. In the future, we are planning to conduct studies to assess the insertion and placement of the implant in detail and studies involving a larger patient population, for more accurate determination of prognostic factors following two-staged bilateral TKR procedures.

In this study we evaluated the inter-procedure similarity of sizes of the implants used during staged surgical treatment for bilateral knee osteoarthritis and identified the prognostic factors involved. This may help better plan surgeries and reduce the risk of complications during the second procedure, which would help achieve improve treatment outcomes and patient satisfaction after the second stage of bilateral knee arthroplasty.

This work will be useful for future researchers as it allows for the identification of prognostic factors when planning and executing a two-staged bilateral TKR. Orthopedists, analyzing the available medical and radiological documentation after the first TKR operation, will be able to predict the number of complications, duration of anesthesia, femoral component size, tibial component size and tibial polyethylene insert size.

## 5. Conclusions

We observed no differences between the two stages of treatment in terms of the duration of anesthesia, duration of hospital stay, femur implant size, tibia implant size, tibial polyethylene insert size, nor the number of complications.

We observed a strong correlation between the size of femoral components used during the first and second total knee arthroplasty. Moreover, we noted a strong correlation between the size of tibial components used during the first and second procedure.

Slightly weaker prognostic factors include the number of complications, duration of anesthesia and tibial polyethylene insert size.

## Figures and Tables

**Table 1:** Characteristics of data from the first and second surgery.

Variable Mean ± Standard Deviation	First Operation	Second Operation	*p*-Value
Duration of hospital stay [days]	6.43 ± 1.98	5.5 ± 1.69	0.211 *
Time of anesthesia during surgery [minutes]	117.04 ± 9.23	118.06 ± 8.29	0.457 *
Femur implant size	5.43 ± 1.46	5.2 ± 1.28	0.54 *
Tibia implant size	5.36 ± 1.55	5.25 ± 1.52	0.382 *
Tibial polyethylene insert size	9.45 ± 0.66	9.34 ± 0.61	0.422 *
Number of complications per patient	0.13 ± 0.34	0.06 ± 0.25	0.371 *

* Wilcoxon signed-rank test; Data are medians and 5th–95th percentiles.

**Table 2:** Correlation between data from the first and second surgery.

Variable	Correlation Coefficient	*p*-Value *
Duration of hospital stay [days]	0.281	0.0640
Time of anesthesia during surgery [minutes]	0.464	0.0014
Femur implant size	0.790	*p* < 0.0001
Tibia implant size	0.820	*p* < 0.0001
Tibial polyethylene insert size	0.379	0.0109
Number of complications per patient	0.418	0.0047

* Spearman’s rank correlation.

**Table 3:** Details data of all patients.

Patient Number	Duration of Hospital Stay [Days]		Femur Implant Size		Tibia Implant Size		Tibial Polyethylene Insert Size		Time of Anesthesia during Surgery [Minutes]		Complications		Order of Surgery	
	First Surgery	Second Surgery	First Surgery	Second Surgery	First Surgery	Second Surgery	First Surgery	Second Surgery	First Surgery	Second Surgery	First Surgery	Second Surgery	First	Second
1	10	7	7	6	7	6	11	9	135	140	1	0	R	L
2	10	10	10	6	7	7	10	9	155	130	1	1	R	L
3	7	4	6	6	7	7	9	9	120	115	0	0	R	L
4	6	5	6	6	7	7	9	9	115	110	0	0	R	L
5	5	4	6	7	6	7	9	9	115	120	0	0	R	L
6	7	4	3	4	2	4	10	9	105	130	0	0	R	L
7	12	10	6	7	4	6	10	10	130	130	1	1	R	L
8	5	6	5	4	5	4	9	9	110	110	0	0	R	L
9	6	7	8	7	8	7	9	10	110	110	0	0	R	L
10	4	4	3	4	4	4	9	9	100	110	0	0	R	L
11	4	5	6	6	6	6	9	9	120	120	0	0	R	L
12	7	4	6	6	6	5	9	9	110	110	0	0	R	L
13	10	7	6	4	3	3	10	9	120	125	1	0	R	L
14	9	7	5	4	5	5	9	9	110	115	1	0	R	L
15	5	6	4	3	4	4	10	9	115	110	0	0	R	L
16	6	7	6	5	6	5	10	11	110	120	0	0	R	L
17	5	6	7	7	6	6	9	9	115	120	0	0	R	L
18	8	4	4	4	4	4	10	10	110	115	0	0	R	L
19	6	4	6	6	7	7	9	9	115	120	0	0	R	L
20	4	5	6	6	6	5	9	9	110	110	0	0	R	L
21	5	6	4	4	4	3	9	9	110	120	0	0	R	L
22	8	6	3	4	4	3	9	9	115	110	0	0	R	L
23	7	5	7	7	8	8	10	9	110	120	0	0	R	L
24	6	7	6	5	6	5	11	10	115	120	0	0	R	L
25	5	6	7	7	6	6	9	9	120	120	0	0	R	L
26	8	4	4	4	6	6	9	9	115	115	0	0	R	L
27	6	4	6	6	7	7	10	10	120	120	0	0	R	L
28	4	5	6	6	7	7	9	9	115	115	0	0	R	L
29	7	6	3	4	3	4	9	9	110	110	0	0	R	L
30	8	4	4	4	3	4	9	11	120	120	0	0	L	R
31	7	8	7	8	8	8	11	9	120	140	0	0	L	R
32	5	5	4	4	4	4	11	11	120	140	0	0	L	R
33	7	9	5	6	5	5	9	9	110	115	0	1	L	R
34	4	6	3	3	4	2	9	9	130	110	0	0	L	R
35	3	5	7	5	7	7	9	10	110	110	0	0	L	R
36	9	4	6	6	6	6	9	9	130	120	1	0	L	R
37	6	4	6	4	7	4	10	9	120	110	0	0	L	R
38	7	4	6	6	5	5	9	9	130	120	0	0	L	R
39	5	4	4	4	3	4	10	10	110	110	0	0	L	R
40	8	6	5	4	4	6	9	10	120	115	0	0	L	R
41	8	7	5	6	6	6	10	10	115	120	0	0	L	R
42	4	5	5	6	5	6	9	9	115	110	0	0	L	R
43	5	3	5	4	4	3	9	9	120	115	0	0	L	R
44	5	3	5	4	4	3	9	9	120	120	0	0	L	R

## Data Availability

The datasets used and/or analyzed during the current study are available from the corresponding author on reasonable request. The data are not publicly available due to privacy.

## References

[1] Sloan M., Premkumar A., Sheth N.P. (2018). Projected Volume of Primary Total Joint Arthroplasty in the U.S., 2014 to 2030. J. Bone Jt. Surg. Am..

[2] Maradit Kremers H., Larson D.R., Crowson C.S., Kremers W.K., Washington R.E., Steiner C.A., Jiranek W.A., Berry D.J. (2015). Prevalence of Total Hip and Knee Replacement in the United States. J. Bone Jt. Surg. Am..

[3] Bedard N.A., Elkins J.M., Brown T.S. (2020). Effect of COVID-19 on Hip and Knee Arthroplasty Surgical Volume in the United States. J. Arthroplast..

[4] Gademan M.G., Hofstede S.N., Vliet Vlieland T.P., Nelissen R.G., de Mheen P.J.M.-v. (2016). Indication criteria for total hip or knee arthroplasty in osteoarthritis: A state-of-the-science overview. BMC Musculoskelet. Disord..

[5] Price A.J., Alvand A., Troelsen A., Katz J.N., Hooper G., Gray A., Carr A., Beard D. (2018). Knee replacement. Lancet.

[6] Chen F., Li R., Lall A., Schwechter E.M. (2017). Primary Total Knee Arthroplasty for Distal Femur Fractures: A Systematic Review of Indications, Implants, Techniques, and Results. Am. J. Orthop..

[7] Wang S., Zhang Y., Li J. (2020). Clinical application of unicompartmental knee arthroplasty and total knee arthroplasty in patient with bilateral knee osteoarthritis. Zhongguo Xiu Fu Chong Jian Wai Ke Za Zhi.

[8] Liu L., Liu H., Zhang H., Song J., Zhang L. (2019). Bilateral total knee arthroplasty: Simultaneous or staged? A systematic review and meta-analysis. Medicine.

[9] Warren J.A., Siddiqi A., Krebs V.E., Molloy R., Higuera C.A., Piuzzi N.S. (2021). Bilateral Simultaneous Total Knee Arthroplasty May Not Be Safe Even in the Healthiest Patients. J. Bone Jt. Surg. Am..

[10] Obaid-ur-Rahman, Hafeez S., Amin M.S., Ameen J., Adnan R. (2021). Unilateral versus simultaneous bilateral total knee arthro-plasty: A comparative study. J. Pak. Med. Assoc..

[11] Grace T.R., Tsay E.L., Roberts H.J., Vail T.P., Ward D.T. (2020). Staged Bilateral Total Knee Arthroplasty: Increased Risk of Recurring Complications. J. Bone Jt. Surg. Am..

[12] Alghadir A.H., Iqbal Z.A., Anwer S., Anwar D. (2020). Comparison of simultaneous bilateral versus unilateral total knee re-placement on pain levels and functional recovery. BMC Musculoskelet. Disord..

[13] Chen J.Y., Lo N.N., Jiang L., Chong H.C., Tay D.K., Chin P.L., Chia S.L., Yeo S.J. (2013). Simultaneous versus staged bilateral unicom-partmental knee replacement. Bone Jt. J..

[14] Pfeil J., Hohle P., Rehbein P. (2011). Bilateral endoprosthetic total hip or knee arthroplasty. Dtsch. Arztebl. Int..

[15] Kim Y.H., Choi Y.W., Kim J.S. (2009). Simultaneous bilateral sequential total knee replacement is as safe as unilateral total knee replacement. J. Bone Jt. Surg. Br..

[16] Memtsoudis S.G., Ma Y., Chiu Y.L., Poultsides L., Gonzalez Della Valle A., Mazumdar M. (2011). Bilateral total knee arthroplasty: Risk factors for major morbidity and mortality. Anesth. Analg..

[17] Scott C.E., Murray R.C., MacDonald D.J., Biant L.C. (2014). Staged bilateral total knee replacement: Changes in expectations and outcomes between the first and second operations. Bone Jt. J..

[18] Ahd J.H., Kang D.M., Choi K.J. (2017). Bilateral simultaneous unicompartmental knee arthroplasty versus unilateral total knee arthroplasty: A comparison of the amount of blood loss and transfusion, perioperative complications, hospital stay, and functional recovery. Orthop. Traumatol. Surg. Res..

[19] Rovňák M., Hrubina M., Šiarnik P., Sýkora J., Melišík M., Nečas L. (2022). Bilateral versus unilateral total knee replacement-comparison of clinical and functional results in two-year follow-up. Rozhl. Chir..

[20] Fabi D.W., Mohan V., Goldstein W.M., Dunn J.H., Murphy B.P. (2011). Unilateral vs. bilateral total knee arthroplasty risk factors increasing morbidity. J. Arthroplast..

[21] Gill S.D., Hatton A., de Steiger R., Page R.S. (2020). One-Surgeon vs. Two-Surgeon Single-Anesthetic Bilateral Total Knee Arthro-plasty: Revision and Mortality Rates From the Australian Orthopedic Association National Joint Replacement Regis-try. J. Arthroplast..

[22] Kazubski K., Tomczyk Ł., Ciszewski M., Witkowski J., Reichert P., Morasiewicz P. (2021). The Symmetry and Predictive Factors in Two-Stage Bilateral Hip Replacement Procedures. Symmetry.

[23] De Luca M.L., Ciccarello M., Martorana M., Infantino D., Letizia Mauro G., Bonarelli S., Benedetti M.G. (2018). Pain monitoring and management in a rehabilitation setting after total joint replacement. Medicine.

[24] Malik A.T., Mufarrih S.H., Ali A., Noordin S. (2019). Predictors of an increased length of stay following Total Knee Arthroplasty-Survey Report. J. Pak. Med. Assoc..

[25] Halawi M.J., Vovos T.J., Green C.L., Wellman S.S., Attarian D.E., Bolognesi M.P. (2015). Preoperative predictors of extended hospital length of stay following total knee arthroplasty. J. Arthroplast..

[26] Song X., Xia C., Li Q., Yao C., Yao Y., Chen D., Jiang Q. (2020). Perioperative predictors of prolonged length of hospital stay following total knee arthroplasty: A retrospective study from a single center in China. BMC Musculoskelet. Disord..

